# Grass Carp Mex3A Promotes Ubiquitination and Degradation of RIG-I to Inhibit Innate Immune Response

**DOI:** 10.3389/fimmu.2022.909315

**Published:** 2022-07-05

**Authors:** Zeyin Jiang, Zhichao Sun, Jihuan Hu, Dongming Li, Xiaowen Xu, Meifeng Li, Zhiqing Feng, Shanshan Zeng, Huiling Mao, Chengyu Hu

**Affiliations:** ^1^ School of Life Science, Key Laboratory of Aquatic Resources and Utilization of Jiangxi Province, Nanchang University, Nanchang, China; ^2^ Human Aging Research Institute, Nanchang University, Nanchang, China; ^3^ Jiangxi Key Laboratory of Human Aging, Nanchang, China; ^4^ School of Basic Medical Sciences, Fuzhou Medical University, Fuzhou, China

**Keywords:** Mex3A, RIG-I, negative regulator, ubiquitination, fish

## Abstract

As one of the Mex3 family members, Mex3A is crucial in cell proliferation, migration, and apoptosis in mammals. In this study, a novel gene homologous to mammalian Mex3A (named *CiMex3A*, MW368974) was cloned and identified in grass carp, which is 1,521 bp in length encoding a putative polypeptide of 506 amino acids. In CIK cells, *CiMex3A* is upregulated after stimulation with LPS, Z-DNA, and especially with intracellular poly(I:C). CiMex3A overexpression reduces the expressions of *IFN1*, *ISG15*, and pro-inflammatory factors *IL8* and *TNFα*; likewise, Mex3A inhibits IRF3 phosphorylation upon treatment with poly(I:C). A screening test to identify potential targets suggested that CiMex3A interacts with RIG-I exclusively. Co-localization analysis showed that Mex3A and RIG-I are simultaneously located in the endoplasmic reticulum, while they rarely appear in the endosome, mitochondria, or lysosome after exposure to poly(I:C). However, RIG-I is mainly located in the early endosome and then transferred to the late endosome following stimulation with poly(I:C). Moreover, we investigated the molecular mechanism underlying CiMex3A-mediated suppression of RIG-I ubiquitination. The results demonstrated that Mex3A truncation mutant (deletion in the RING domain) can still interact physically with RIG-I, but fail to degrade it, suggesting that Mex3A also acts as a RING-type E3 ubiquitin ligase. Taken together, this study showed that grass carp Mex3A can interact with RIG-I in the endoplasmic reticulum following poly(I:C) stimulation, and then Mex3A facilitates the ubiquitination and degradation of RIG-I to inhibit IRF3-mediated innate antiviral immune response.

## Highlights

1. CiMex3A is a negative regulator in innate immunity.
2. CiMex3A interacts with RIG-I.
3. CiMex3A and RIG-I are located in the endoplasmic reticulum.
4. CiMex3A promotes RING domain-mediated ubiquitination and degradation of RIG-I.

## Introduction

Innate immunity exerts a crucial role in the first line of host defenses against bacteria, viruses, and many other pathogenic organisms (1–3). Pattern recognition receptors (PRRs) are indispensable in triggering innate immune responses in organisms. PRRs can fall into several categories including Toll-like receptors, RIG-I-like receptors, NOD-like receptors, and C-type lectin receptors (4–6). The RIG-I-like receptor family has fewer members than the Toll-like receptors but is critical in antiviral innate immunity due to its role in recognizing foreign RNA in the cytoplasm (7–9). For example, upon treatment with double-stranded RNA (dsRNA) or single-stranded RNA (ssRNA), RIG-I recruits mitochondrial antiviral signaling (MAVS) protein to activate TNK-binding kinase 1(TBK1) (10), followed by TBK1-induced IRF3 phosphorylation, and eventually resulting in the expression of type I interferon (IFN1) (11, 12).

There are three members in the RIG-I-like receptor (RLR) family, i.e., RIG-I, LGP2, and melanoma differentiation-associated gene 5 (MDA5) (13, 14), and each of them contains a similar DExD/H-Box helicase domain (15). In mammals, RIG-I is involved in the activation of IRF-3 and NF-κB (16). At present, a great number of studies demonstrate that many proteins regulate the RIG-I antiviral pathway. For instance, TRIM25 can induce Lys63-linked ubiquitination of RIG-I to initiate an antiviral immune response in mammals (17). USP27X negatively regulates innate immunity through the deubiquitination of RIG-I (18). Zinc-finger ZCCHC3 facilitates RIG-I-like receptor-mediated innate immunity response (19).

Nowadays, an increasing number of RLRs have been cloned and identified in teleosts, such as grass carp (20), common carp (21), and Atlantic salmon (22). Zebrafish RIG-I enhances RIG-I/MAVS-mediated signaling and results in a higher level of antiviral response in defense against SVCV infection (23). Lai et al. showed that fish RIG-I is strongly related to ubiquitination processes (24).

Mex3A, a member of the RNA-binding protein Mex3 family (25), is first identified in human (26). Mex3A contains two K homologous domains (KH) at the N-terminus and a Ring finger domain at the C-terminus with E3-ligase activity (26–28). Mex3A plays a vital role in the development of many human cancers. For example, Mex3A promotes the proliferation and migration of triple-negative breast cancer (TNBC) cells through the PI3K-AKT signaling pathway (25), interacts with RAP1GAP in CRC cells to promote oncogenesis in colorectal cancer (29), or promotes adenocarcinoma metastasis by interaction with LAMA2 (30). In addition, Mex3A also participates in apoptosis (31). However, there are a few studies regarding the antiviral properties of mammalian Mex3A in innate immunity at present.

It is widely known that innate immunity is extremely important for lower aquatic vertebrate such as fish to resist viral infections (32). Up to now, there have been few studies about Mex3A-mediated innate immunity in fish. Baumgart et al. demonstrated that zebrafish Mex3A is expressed in embryos (33). Naef et al. revealed that zebrafish Mex3A is a regulator of neurogenesis in the central nervous system (34).

In this study, grass carp Mex3A (CiMex3A) was cloned and identified. The results showed that CiMex3A negatively regulates innate immune response *via* the ubiquitination and degradation of RIG-I.

## Materials and Methods

### Grass Carp and Cell Lines

Grass carp were obtained from Nanchang Shenlong Fisheries Development (Jiangxi, China) and domesticated in our laboratory. The breeding conditions were described in our previous study (35). CIK (*C. idella* kidney) cells and CO (*C. idella* Overy) cells from Professor Pin Nie (Institute of Hydrobiology, Chinese Academy of Sciences) were applied in the experiments. Both CIK cells and CO cells needed Medium 199 (CORNING, USA), to which was added 10% fetal bovine serum (FBS) and 0.6% penicillin–streptomycin liquid (Beijing Solarbio Science & Technology, China); the mixture was then cultured at 28°C in a cell incubator. In addition, CO cells require 5% CO_2_.

### Cloning of Grass Carp Mex3A

CIK cells were cultured in 6-well plates (Wuxi NEST Biotechnology Co., Ltd., China) for 24 h. After the medium was removed, the cells were washed three times with PBS. Total RNA was extracted using the RNA simple Total RNA Kit (Tiangen Biotech, China). The concentration and quality of total RNA were measured by a QuickDrop Spectrophotometer (USA) (OD_260_/OD_280_ > 2.0). The samples were tested in agarose gel electrophoresis. Three electrophoretic bands (5S, 18S, and 28S) were clearly shown. cDNA from about 1 μg of total RNA was synthesized using the PrimeScript RT Reagent Kit with gDNA Eraser Perfect Real Time (TaKaRa Bio, China). cDNA dilution was used as the template for PCR amplification of Mex3A. The primers (**Table 1**) were designed employing an Oligo7 program according to *D. rerio* Mex3A sequence (XM_009292667.3). PCR amplification programs were as follows: pre-denaturation at 95°C for 5 min, 35 cycles of 95°C denaturation for 30 s, 53°C annealing for 30 s, and 72°C elongation for 2 min, with a final elongation at 72°C for 10 min. The PCR product was purified using the Sanprep Column PCR Product Purification Kit (Sangon Biotech, China). Finally, the purified PCR product was directly inserted into pEASY-T1 vectors (TaKaRa Bio, China) and sequenced (Shanghai Sangon, China). The sequence analysis was conducted by Nucleotide BLAST of NCBI (http://www.ncbi.nlm.nih.gov/blast). The domain of grass carp Mex3A (CiMex3A) was predicted by SMART (http://smart.embl.de/).

**Table 1:** Sequences and application of primers and siRNA used in this study.

Primer name	Primer sequence (5’-3’)	Application
Mex3A-ORF-F	ATGCCTAGTTTGCTGGTT	ORF
Mex3A-ORF-R	TTAAGAGAATATTCGTATAGCCTG	
Mex3A-(1-816)-F- GFP**-**BGLII	GAAGATCTATGCCTAGTTTGCTGGTT	Eukaryotic Vector construction
Mex3A-(1-816)-R-GFP-ECORI	CCGGAATTCCGCCTCGATTTCCTCCC	
Mex3A-pcDNA3.1-F-ECORI	CCGGAATTCATGCCTAGTTTGCTGGTT	
Mex3A-pcDNA3.1-R-XhoI	CCGCTCGAGTTAAGAGAATATTCGTATAGCCTG	
Mex3A-(1-816)-pcDNA3.1-F-BGLII	GAAGATCTATGCCTAGTTTGCTGGTT	
Mex3A-(1-816)-pcDNA3.1-R-XhoI	CCGCTCGAGCGCCTCGATTTCCTCCC	
Mex3A-(1-1362)-pcDNA3.1-F-BGLII	GAAGATCTATGCCTAGTTTGCTGGTT	
Mex3A-(1-1362)-pcDNA3.1-R-XhoI	CCGCTCGAGGTCCCGAGCCACGGCAG	
Mex3A-(1-1362)-GFP-F-BGLII	GAAGATCTATGCCTAGTTTGCTGGTT	
Mex3A-(1-1362)-GFP-R-ECORI	CCGGAATTCGTCCCGAGCCACGGCAG	
Mex3A-GFP-F-BGLII	GAAGATCTATGCCTAGTTTGCTGGTT	
Mex3A-GFP-R-ECORI	CCGGAATTCTTAAGAGAATATTCGTATAGCCTG	
Mex3A-Flag-F-ECORI	CCGGAATTCAATGCCTAGTTTGCTGGTT	
Mex3A-Flag-R- BGLII	GAAGATCTTTAAGAGAATATTCGTATAGCCTG	
RIG-I-GFP-F-BGLII	GAAGATCTATGTACGAGCTGGAAAAGGAGA	
RIG-I-GFP-R-ECORI	CCGGAATTC TCAGTCTCTCAGCGGCCA	
RIG-Flag-F-ECORI	CCGGAATTCAATGTACGAGCTGGAAAAGGAGA	
RIG-Flag-R-BGLII	GAAGATCTTCAGTCTCTCAGCGGCCA	
PRMT6-GFP-BGLII-F	GAAGATCTATGGCAAACTTGGGGAA	
PRMT6-GFP-Hind III-R	CCCAAGCTTGGCCAAACTTCTATTTAACTTC	
RAB5-F-HindIII-RED	CCCAAGCTTCGATGGCAGGAAGAGGTGGA	
RAB5-R-EcoRI-RED	CCGGAATTCTTAGTTGCTACAGCAGGG	
RAB7-F-EcoRI-RED	CCGGAATTCTATGACATCAAGGAAGAAAGTTC	
RAB7-R-KPNI-RED	CGGGGTACCTCAGCAGCTACAAGTCTCTG	
TLR3-GFP-XhoI-F	CCGCTCGAGCTATGGAACTGATGAAACTCATACTG	
TLR3-GFP-ECORI-R	CCGGAATTCCTAATTAACCTTGTTGGAAGAGGC	
Mex3A-RT-F	ATGCCTAGTTTGCTGGTTCTA	QRT-PCR
Mex3A-RT-R	CGATGGGATGTGATTGTGGTG	
IFN1-RT-F	GTCAATGCTCTGCTTGCGAAT	
IFN1-RT-R	CAAGAAACTTCACCTGGTCCT	
ISG15-RT-F	TGTTGAACGGAGATGTGAGGT	
ISG15-RT-R	TAACTGCTGAGGCTTCTGGAA	
MX2-RT-F	ACATTGACATCGCCACCACT	
MX2-RT-R	TTCTGACCACCGTCTCCTCC	
IL8-RT-F	CCCTACTGCTCCCTGGGTTA	
IL8-RT-R	CCAAGCAGAATGGTGCAGGT	
TNFα-RT-F	GCTGCTGTCTGCTTCACGC	
TNFα-RT-R	AGCCTGGTCCTGGTTCACTCT	
β-actin-F	CACTGTGCCCATCTACGAG	
β-actin-R	CCATCTCCTGCTCGAAGTC	
Si-Mex3A-sense	GGAUGUUACAAGGGAAGAATT	Knockdown
Si-Mex3A-antisense	UUCUUCCCUUGUAACAUCCTT	
NC (negative control)	UUCUCCGAACGUGUCACGUTT	

### Plasmids Construction

The ORF of *CiMex3A* was separately inserted into pcDNA3.1 (+) (Invitrogen, USA), pEGFP-C1 (Invitrogen, USA), and p3×FLAG-Myc-CMV™-24 (Invitrogen, USA) plasmids. The mutants Mex3A (1–282) and Mex3A (1–454) were inserted into pcDNA3.1 (+) (Invitrogen, USA) and pEGFP-C1 (Invitrogen, USA), respectively. The ORF of RIG-I (GQ478334.2) was separately constructed into p3×FLAG-Myc-CMV™-24 and pEGFP-C1. The ORFs of *TRIF* (KC333648.1), *PRMT6* (MN781672.1), and *TLR3* (DQ864497.1) were separately inserted into pEGFP-C1. CDS regions of grass carp RAB5 (MF598473.1) and RAB7 (MF598474.1) were separately constructed into pDsRed2-C1 (Sangon Biotech, China). PKZ-GFP (36), ZDHHC1-GFP (37), TRAF6-GFP (38), pDsRED2-ER, and pDsRED2-Mitoplasmids (Biofeng) were stored in our lab. All the primers for vector construction are listed in **Table 1**.

### Cell Transfection and Gene KnockDown Assays

CIK or CO cells were seeded into 6-well culture plates (Wuxi NEST Biotechnology Co., Ltd., China) or confocal dishes. After 12 h, the cells were transfected with plasmids as indicated using Lipo8000™ Transfection Reagent (Beyotime Biotechnology, China) according to the manufacturer’s instructions. SiRNA against CiMex3A or NC (negative control) was designed and synthesized by Suzhou GenePharma. CIK cells and CO cells were separately seeded into 6-well culture plates, and 12 h later, the cells were transfected either with 5 μl of *NC* or with 5 μl of *siRNA* using an equal volume of LipoRNAi™ Transfection Reagent (Beyotime Biotechnology, China). The sequences of *NC* and *siRNA* are listed in **Table 1**.

### Quantitative Real-Time PCR

CIK cells were seeded into six-well plates. After 12 h, the cells were transfected with 2.0 μg of pcDNA3.1-basic or Mex3A-pcDNA3.1 plasmid using 3.2 μl of Lipo8000™ Transfection Reagent (Beyotime Biotechnology, China) in one group. The cells were transfected with poly(I:C) (Sigma, USA) in another group. CIK cells were transfected with 5 μl of *NC* or 5 μl of *siRNA* against CiMex3A. Then, total RNA was extracted using an RNA extraction kit as described above. Analyses of expression profiles of *IFN1*, *ISG15*, *MX2*, *IL8*, and *TNFα* were carried out on CFX Connect™ (Bio-Rad, USA). *β-actin* was used as an internal control.

### Determination of Subcellular Localization Using an Immunofluorescence Approach

CIK cells were cultured in microscopic petri dishes for 12 h, and then the cells were separately transfected with 1.0 μg of Mex3A-GFP, 1.0 μg of Mex3A (1–282)-GFP, and 1.0 μg of Mex3A (1–454)-GFP. After 24 h, the medium was removed and then the cells were gently washed three times with PBS, followed by fixation with 4% paraformaldehyde (Bio-Medical Assay Co., Ltd., China) at room temperature. Fifteen minutes later, the cells were dyed with DAPI (Sangon Biotech, China) and then kept in darkness for 15–20 min. Finally, the samples were washed three times with PBS and images were documented using a confocal microscope (Confocal microscope LSM800, Zeiss, Germany).

CIK cells were co-transfected with 0.5 μg of Mex3A-GFP and 0.5 μg of RIG-I-Flag plasmids. Twenty-four hours later, the cells were washed three times with PBS and fixed with 4% paraformaldehyde. The cells were permeabilized using 0.3% Triton X-100, then blocked with 5% bovine serum albumin, and then kept at room temperature for 1 h, followed by incubation of FLAG-tag antibody (Sigma, USA, F1804) overnight at 4°C. FLAG-tag antibody was recovered, and the cells were washed three times, 10 min each time. Moreover, the cells were incubated with secondary antibody goat anti-mouse Cy3 (BioLegend, USA) at room temperature for 2 h. Next, the cells were counterstained with DAPI for 15–20 min and photographs were taken using a laser confocal microscope (Zeiss, Germany).

To determine whether CiMex3A is co-located with Lyso-Tracker (Beyotime Biotechnology, China), CIK cells were separately transfected with Mex3A-GFP or RIG-I-GFP. Twenty-four hours later, the cells were stimulated with poly(I:C) for 6 h. In addition, the cells were incubated with the above markers according to the manufacturer’s instructions. RAB5-pDsRed2, RAB7-pDsRed2, pDsRED2-ER, and pDsRED2-Mitoplasmids were used to trace the interactions between Mex3A and RIG-I in the early endosome, late endosome, mitochondrion, and endoplasmic reticulum, respectively. The immunofluorescence co-localization analysis was performed using the experimental operations as described above.

### Western Blotting

CO cells were used to explore the binding of Mex3A to some potential substrate targets. First, CO cells were seeded into 10-cm petri dishes (Wuxi NEST Biotechnology Co., Ltd., China). After 12 h, the cells were separately co-transfected with Mex3A-flag and each of the following recombined plasmids: PKZ-GFP, TRAF6-GFP, IRF7-GFP, PRMT6-GFP, ZDHHC1-GFP, TRIF-GFP, RIG-I-GFP, and TLR3-GFP. At 24 h post transfection, 1 ml of NP40 lysis buffer (Beyotime Biotechnology, China) containing 1 mM phenylmethyl sulfonyl fluoride (PMSF, Sangon Biotech, China), 1 μg/ml leupeptin (Sangon Biotech, China), 1 μg/ml aprotinin (Sangon Biotech, China) and 1 mM phosphate inhibitor cocktail (CWBIO, Beijing, China) was applied to lyse the cells in one 10-cm petri dish on ice for 30 min. Then, it was transferred to a new 1.5-ml centrifuge tube and centrifuged at 4°C for 10 min. The supernatant was transferred to another new tube. Then, 20 μl of 5× protein loading buffer was added into 100 μl of supernatant and boiled at 95°C for 10 min. The remaining supernatant was equally divided into two groups, one group of which was added with 3 μl of mouse IgG antibody (Sangon Biotech, China), while the same amount of anti-FlagM2Ab (Sigma) was added to the other group. The sample was incubated at 4°C for 2 h. Then, 25 μl of anti-mouse nanobody IP agarose beads (Shenzhen Health Life Technology Co., Ltd) was added into the above supernatant and incubated for 3 h. Furthermore, the mixture was centrifuged at 4°C. The supernatant was removed and mixed with 500 μl of wash buffer (10 mM Tris-HCl, pH 7.5; 150 mM NaCl; 0.5 mM EDTA). Agarose beads were collected by centrifugation. The experiment as described previously was repeated three times; 50 μl of 2× loading buffer was added to agarose beads and boiled at 95°C for 10 min. The above-mentioned protein samples were separated through SDS-PAGE electrophoresis. Then, they were transferred to nitrocellulose membranes (Millipore, USA). The membranes were blocked with 5% non-fat milk for 1 h and incubated in specific antibody at 4°C overnight. The next day, the membranes were incubated with the indicated secondary antibody at room temperature for 1 h. Lastly, the membranes were examined using a chemiluminescence imaging system (CLINX, China).

CO cells were transfected with 1.0 μg of Mex3A-pcDNA3.1. At 6 h post stimulation with poly(I:C), IRF3 phosphorylation (IRF3-p Beyotime Biotechnology, China) was detected. The basal IRF3 antibody (Biotechnology, China) and GAPDH antibody (reserved in our lab) served as internal controls. IFN1 antibody was also reserved in our lab. In addition, GFP-Tag antibody (Shanghai Abmart Co., Ltd) and Anti HA-Tag (Jiangsu, CWBIO) were used in the ubiquitination assay.

### Statistical Analysis

Data were analyzed as mean ± SD. Statistical tests were confirmed by the Student’s *t*-test and ANOVA. The analyses were conducted using Graphpad Prism Version 6.0. The level of significance was measured as *p < 0.05 or **p < 0.01.

## Results

### Phylogenetic Characteristics and Expression Analysis of Grass Carp Mex3A

*CiMex3A* (MW368974) is 1,521 bp in full length, which encodes a putative protein of 506 amino acids. Mex3A protein sequences from different species are used to generate a phylogenetic tree. The results indicated that grass carp Mex3A shares the highest level of sequence similarity with that of *D. rerio* (**Figure S1**).

**Figure 1:**
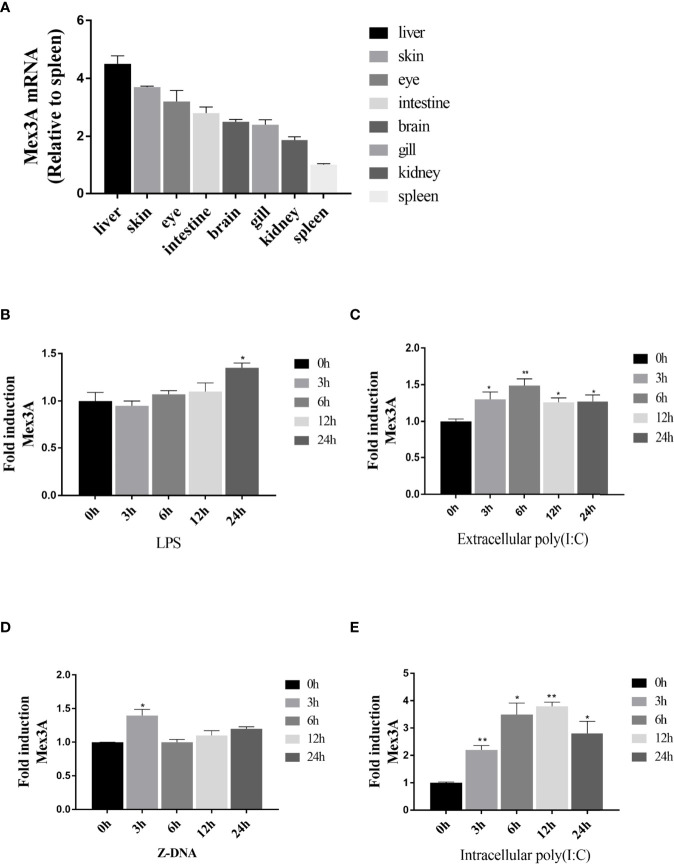
Expression of grass carp Mex3A. **(A)** Constitutive expression of grass carp Mex3A in healthy fish tissues; *β-actin* was used as an internal control. **(B, C)** CIKs were cultured and incubated in 6-well plates for 12 h, and then the cells were separately treated with LPS (2 μg/ml) or poly(I:C) (2 μg/ml). Then, the expression of Mex3A was detected by qRT-PCR. **(D, E)** CIK cells were separately transfected with Z-DNA or poly(I:C) (2 μg/ml). Mex3A expression was determined by qRT-PCR, *β-actin* was used as an internal control. Data are presented as mean ± SD (*n* = 3). *p < 0.05, **p < 0.01.

*CiMex3A* is extensively expressed in various tissues of grass carp. The expression level of *CiMex3A* is relatively higher in the liver and skin when compared with other tissues (**Figure 1A**). In addition, *CiMex3A* expression was unexceptionally upregulated under stimulation with LPS at 24 h (**Figure 1B**), with extracellular poly(I:C) peaking at 6 h (**Figure 1C**), and with Z-DNA at 3 h (**Figure 1D**). The expression level of *CiMex3A* hit its peak at 12 h following treatment with intracellular poly(I:C) (**Figure 1E**).

### Grass Carp Mex3A Acts as a Negative Regulator in Innate Immunity

**Figure 2:**
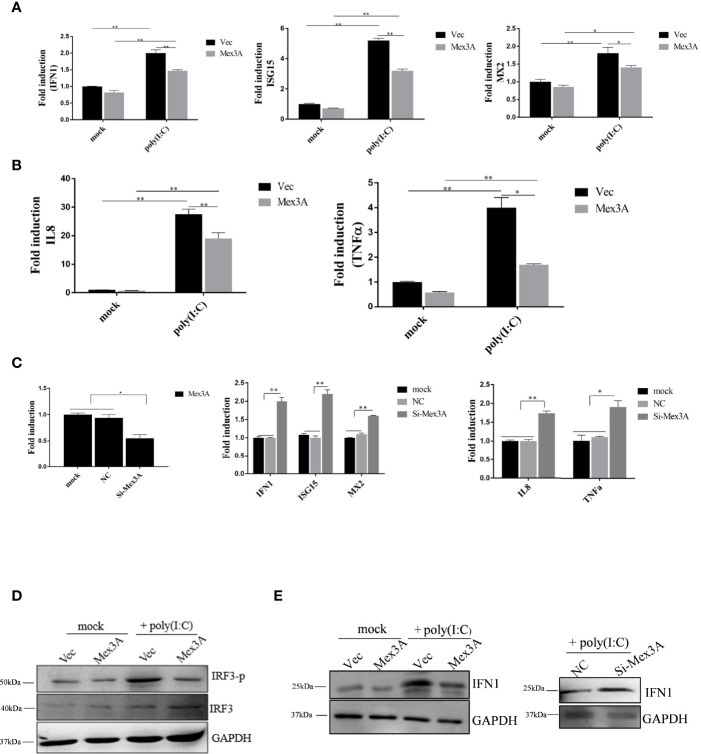
Grass carp Mex3A acts as a negative regulator in innate immunity. **(A)** CIK cells were separately transfected with 2.0 μg of pcDNA3.1-basic or 2.0 μg of Mex3A-pcDNA3.1 plasmid. Six hours later, the expressions of *IFN1*, *ISG15*, *MX2*, were analyzed by qRT-PCR. *β-actin* was used as an internal control. **(B)** CIK cells were transfected with the indicated plasmids. Expressions of *IL8* and *TNFα* were measured and analyzed. **(C)** CIK cells were separately transfected with 5 μl of NC and 5 μl of Si-Mex3A. At 24 h post transfection, the expressions of *Mex3A*, *IFN1*, *ISG15*, *MX2*, *IL 8*, and *TNFα* were tested. **(D)** CIK cells were separately transfected with the indicated plasmids, and the phosphorylation level of IRF3 was detected by Western blotting. **(E)** The cells were transfected with the indicated plasmids, Western blot was performed with IFN1 antibody, and GAPDH antibody was used as an internal control. The results are representative of three independent experiments. Data are analyzed with ANOVA and presented as mean ± SD. *p < 0.05, **p < 0.01.

To explore the role that Mex3A plays in antiviral innate response, the overexpression of Mex3A was carried out in CIK cells. The results showed that the expression levels of *IFN1*, *ISG15*, and *MX2*, as well as proinflammatory cytokines *IL8* and *TNFα* are all downregulated, and it is particularly obvious at 6 h post stimulation with poly(I:C) (**Figures 2A, B**). However, after RNAi-mediated knockdown of Mex3A, their expressions were upregulated at the mRNA level (**Figure 2C**). Furthermore, CiMex3A reduced the phosphorylation of IRF3 (**Figure 2D**) and the production of IFN1 (**Figure 2E**). These results demonstrated that CiMex3A can suppress innate antiviral response when the cells were stimulated with poly(I:C), indicating that CiMex3A can attenuate poly(I:C)-induced innate immune response.

### Grass Carp Mex3A Interacts With RIG-I

**Figure 3:**
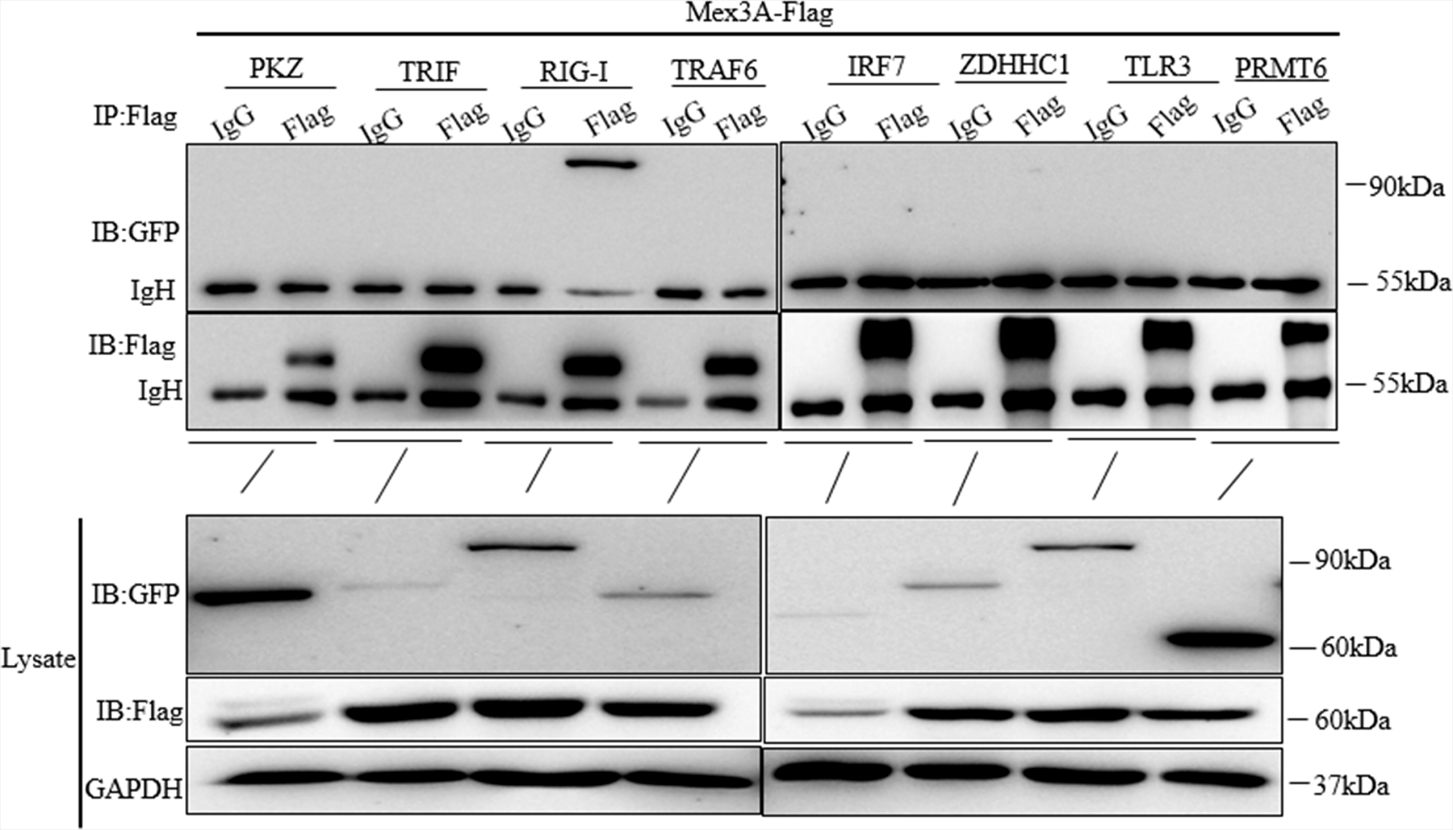
Grass carp Mex3A interacts with RIG-I. CO cells were individually co-transfected with Mex3A-Flag plasmids and PKZ-GFP, TRIF-GFP, RIG-I-GFP, TRAF6-GFP, IRF7-GFP, ZDHHC1-GFP, TLR3-GFP, and PRMT6-GFP. Western blotting was used to analyze all the immunoprecipitation samples. The results are representative of three independent experiments.

To further investigate the role that Mex3A plays in host antiviral innate response, it is critical to identify the Mex3A-dependent signal pathway. Mex3A-Flag was individually co-transfected with PKZ-GFP, TRIF-GFP, RIG-I-GFP, TRAF6-GFP, IRF7-GFP, ZDHHC1-GFP, TLR3-GFP, and PRMT6-GFP. Immunoprecipitation and Western blotting demonstrated that Mex3A can interact with RIG-I (**Figure 3**), indicating that Mex3A may participate in the RIG-I-mediated signal pathway.

### Grass Carp Mex3A and RIG-I Are Located in the Endoplasmic Reticulum

**Figure 4:**
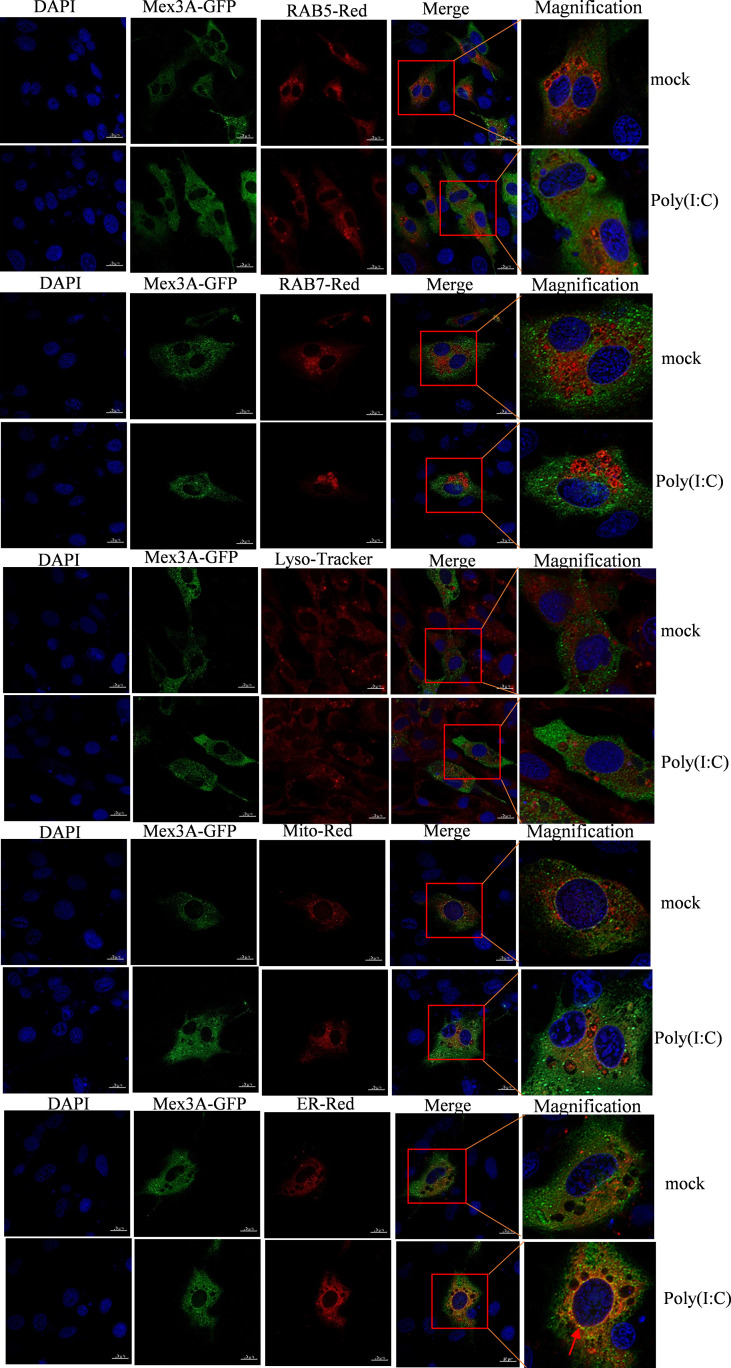
Grass carp Mex3A is located in endoplasmic reticulum. CIK cells were separately co-transfected with 0.5 μg of Mex3A-GFP and 0.5 μg of RAB5-pDsRed2, as well as 0.5 μg of RAB7-pDsRed2, 0.5 μg of Mito-pDsRed2, or 0.5 μg of ER-pDsRed2 plasmids. The photographs were documented using confocal microscopy. In addition, CIK cells were transfected with 1 μg of Mex3A-GFP. At 24 h post transfection, the cells were incubated with Lysosome-Tracker. The bars show 10μm.

The subcellular localization of Mex3A and RIG-I was investigated in CIK cells under stimulation with poly(I:C). The results showed that CiMex3A is rarely located either in the early endosome marker RAB5 or in the late endosome marker RAB7; likewise, CiMex3A is rarely co-located with RIG-I in the lysosome. However, Mex3A was partially located in the endoplasmic reticulum under stimulation with poly(I:C) (**Figure 4**). RIG-I was mainly located in the early endosome RAB5 but rarely in the late endosome RAB7 before the cells were stimulated with poly(I:C). However, RIG-I is particularly accumulated and located in late endosome RAB7 upon poly(I:C) stimulation. In addition, RIG-I was partially located in the mitochondria and endoplasmic reticulum, but rarely in the lysosome (**Figure S2**). The results showed that Mex3A and RIG-I are located in the endoplasmic reticulum following stimulation with poly(I:C).

### Grass Carp Mex3A Promotes Ubiquitination of RIG-I

**Figure 5:**
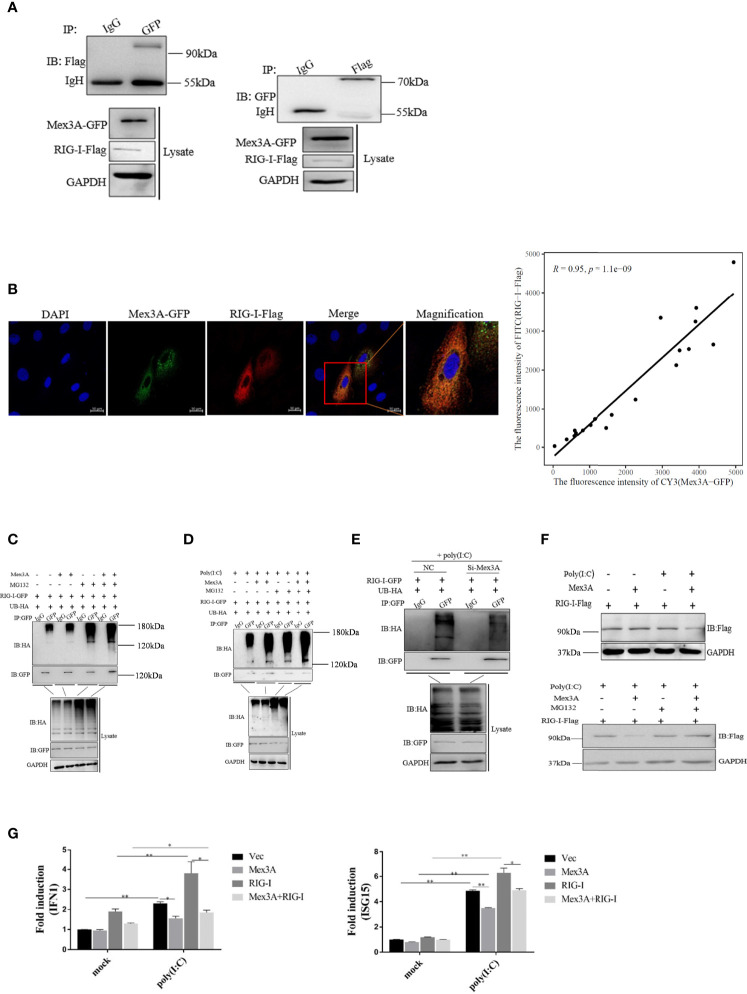
Grass carp Mex3A promotes ubiquitination of RIG-I. **(A)** The interaction of Mex3A and RIG-I was analyzed with Western blotting. **(B)** Co-localization of Mex3A and RIG-I was examined in CIK cells. The photographs were taken on confocal microscope. Furthermore, the co-localization ratio is analyzed using Pearson′s coefficient analysis. The bars show 10μm **(C)** The ubiquitination of RIG-I was analyzed by immunoprecipitation with anti-HA, followed by Western blotting analysis using the indicated antibodies. **(D)** The ubiquitination of RIG-I was still analyzed by immunoprecipitation and tested by Western blotting. **(E)** CO cells were separately transfected with 10 μl of *NC* or 10 μl of *Si-Mex3A*. The cells were treated with poly(I:C) for 6 h before ubiquitination of RIG-I was analyzed. **(F)** CO cells were transfected with indicated plasmids and were treated with poly(I:C) or MG132. Cells were lysed and the samples were analyzed by Western blotting with anti-Flag antibody; GAPDH antibody is used as the internal control. **(G)** The cells were transfected with the indicated plasmids. The expressions of *IFN1* and *ISG15* were analyzed by qRT-PCR like in **Figure 1A**. The results are from three independent experiments. Data are analyzed with ANOVA and presented as mean ± SD (*n* = 3). *p < 0.05, **p < 0.01.

Grass carp Mex3A is found to interact with RIG-I (**Figure 5A**). Subcellular localization assays also demonstrated that Mex3A is co-located with RIG-I in the cytoplasm and the co-localization ratio is 0.95 (**Figure 5B**). RIG-I-GFP and UB-HA plasmids were expressed in CO cells, and ubiquitination of RIG-I can be obviously observed. Following treatment with MG132 (a proteasome inhibitor), the ubiquitin conjugation of RIG-I was increased (**Figure 5C**). More importantly, when the cells were treated with poly(I:C), Mex3A promoted the ubiquitination of RIG-I. The results suggested that Mex3A participates in the proteasome-dependent degradation of RIG-I (**Figure 5D**). Furthermore, after Mex3A was knocked down, the cells were treated with poly(I:C), and the ubiquitination of RIG-I was also decreased (**Figure 5E**). The degradation of RIG-I was enhanced in the presence of Mex3A, especially when the cells were treated with poly(I:C). Moreover, MG132 inhibited the degradation of RIG-I (**Figure 5F**). The results further revealed that Mex3A promotes the ubiquitination and degradation of RIG-I.

Additionally, it was found that the mRNA levels of *IFN1* and *ISG15* were reduced when the cells were co-transfected with Mex3A-pcDNA3.1 and RIG-I-pcDNA3.1 (**Figure 5G**).

In summary, Mex3A negatively regulates innate immunity *via* the ubiquitination and degradation of RIG-I.

### Grass Carp Mex3A Promotes Ubiquitination of RIG-I by the RING Domain

**Figure 6:**
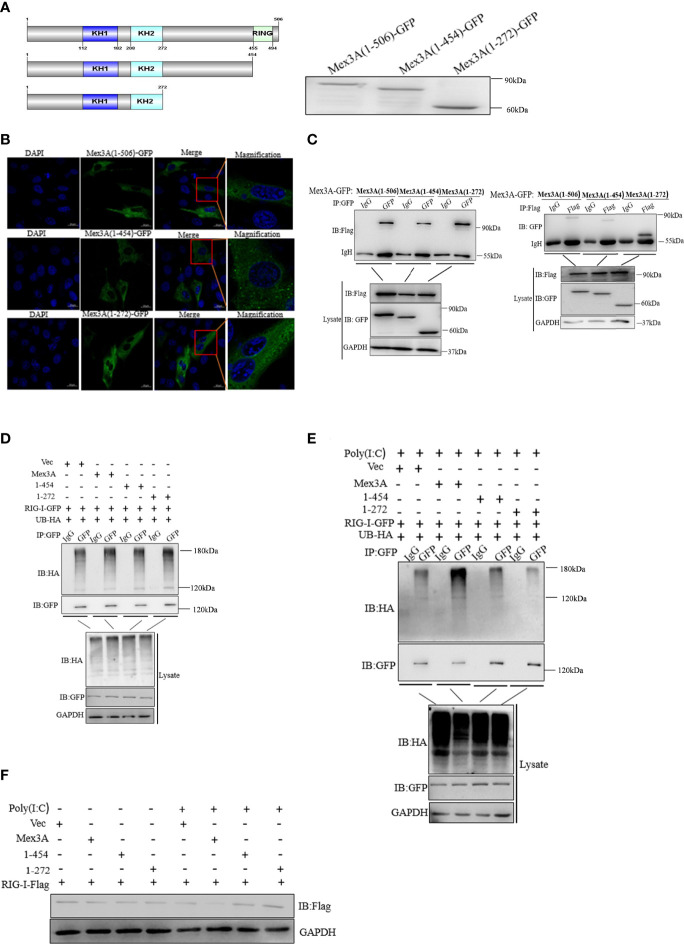
Mex3A promotes ubiquitination of RIG-I by the RING domain. **(A)** A schematic presentation and expression on full-length Mex3A and its mutants. **(B)** Subcellular localization analysis of Mex3A and its mutants. The samples were tested and the photographs were taken under 63× oil immersion using a Zeiss confocal microscope. The bars show 10μm **(C)** Domain mapping of the Mex3A and its mutants with RIG-I. The CO cells were transfected with either Mex3A-GFP or the indicated Mex3A mutant plasmids (2.5 μg) and RIG-I-Flag plasmids (2.5 μg). Co-immunoprecipitation and Western blotting were performed with the indicated antibodies. **(D, E)** CO cells were transfected with the indicated plasmids. The cells were treated with poly(I:C) for 6 h in panel **(E)** The ubiquitination of RIG-I was analyzed by immunoprecipitation with anti-HA, followed by Western blotting analysis using the indicated antibodies used in **Figures 4C, D**. **(F)** The full-length of Mex3A promotes the degradation of RIG-I. CO cells were transfected with the indicated plasmids. The cells were lysed and analyzed by Western blotting with anti-Flag antibody; GAPDH antibody was used as the internal control. The results are based on three independent experiments.

To verify the mechanism underlying Mex3A-mediated ubiquitination and degradation of RIG-I, two Mex3A truncation mutants were constructed, i.e., Mex3A (1–272) mutant containing K homology (KH) domain and Mex3A (1–454) mutant (deletion in the RING domain) (**Figure 6A**).

Mex3A and its mutants were located in the cytoplasm (**Figure 6B**). CO cells were separately co-transfected with RIG-I-Flag and Mex3A (1–272)-GFP as well as Mex3A (1–454)-GFP. The results indicated that Mex3A without the RING domain can also interact with RIG-I (**Figure 6C**). CO cells were individually transfected with UB-HA, RIG-I-GFP, and Mex3A truncation mutants 1–272-pcDNA3.1 and 1–454-pcDNA3.1. Overexpression of these mutants failed to increase the ubiquitination of RIG-I (**Figures 6D, E**). The results showed that Mex3A promotes the ubiquitination of RIG-I *via* the RING domain. Correspondingly, the degradation of RIG-I also relies on its RING domain in Mex3A (**Figure 6F**).

## Discussion

PRRs are essential to recognize viral PAMPs and limit their replications (39, 40). Post-translation modifications (PTMs) modulate the activity and function of PRRs (4). The main forms of PTMs, such as phosphorylation, polyubiquitination, methylation, acetylation, SUMOylation, and succinylation, can regulate innate immunity, inflammation, and other kinds of cellular physiological reactions through interaction with receptors, adaptors, and signaling molecules (4, 5). RIG-I is a key PRR that can exert an important role in detecting virus-derived RNAs in the cytoplasm (41). At present, many studies report that RIG-I has many types of post-translational modifications. For instance, HDAC6 can transiently interact with RIG-I and remove its lysine 909 acetylation in the presence of viral RNAs, which promotes RIG-I to detect RNAs through its C-terminal region (42). IKKϵ interacts with and then phosphorylates RIG-I to regulate NF-κB and IFN-β (43). Small ubiquitin-like modifier-1 (SUMO-1) affects RIG-I SUMOylation, which enhances IFN-I production (44). In addition, RIG-I can be activated by other ubiquitination pathways. For instance, RNF122 can catalyze K48-linked ubiquitination and finally promote the degradation of RIG-I (40). E3 ubiquitin ligase c-Cbl catalyzes the attachment of K48-linked ubiquitination to RIG-I, leading to the degradation of RIG-I (45). Mex3C interacts with RIG-I and causes lysine-63-linked ubiquitination of RIG-I, resulting in the higher production of type I IFN (46). Although RNF122, c-Cbl, and Mex3C belong to different categories, they are members of the E3 ubiquitin ligase family, and the molecular mechanism underlying the same substrate catalyzed by different E3 ligases is extremely variable (40). Here, we identified a member of the Mex3 family in grass carp, namely, CiMex3A, which is a RNA-binding protein with a RING domain (47).

*CiMex3A* was widely expressed in different tissues of grass carp, especially in the liver (**Figure 1A**). In mammals, *Mex3A* is upregulated in liver cancer and used as the prognostic factor of liver cancer (48). In this paper, we showed that CiMex3A expression was increased under stimulation with poly(I:C) (**Figure 1E**), indicating that CiMex3A may participate in the signal pathway activated by cytoplasmic signal molecules. Many previous studies showed that MDA5, one member of RIG-I-like receptors, can be activated by cytoplasmic double-stranded RNA (49, 50). Furthermore, RIG-I can recognize cytoplasmic viral RNA (40) or cytoplasmic poly(I:C) (51). We speculate that fish Mex3A may be involved in the cytoplasmic receptor-mediated signal pathway. However, apart from cytoplasmic and extracellular poly(I:C), LPS and Z-DNA also enhanced the expression of CiMex3A, but the efficacy is not very significant in comparison with that of cytoplasmic poly(I:C) (**Figures 1B–E**). LPS participates in the signaling pathway mediated by TRAF6 in grass carp (38). Z-DNA is the essential stimulation in the ZDHHC1-STING-mediated innate immunity pathway (37). Extracellular poly(I:C) affects the TLR3-mediated signaling pathway (52). Certainly, fish Mex3A may fall into the signal pathway activated by those pathogen-associated molecular mechanisms, but the regulatory mechanism needs to be further explored.

Mex3 family is involved in RNA metabolism (26, 28). In our study, CiMex3A is a negative factor in regulating antiviral innate immunity and inflammatory response (**Figure 2**). However, in a previous study, it is very interesting to find that, as an E3 ubiquitin ligase member, both RNF122 and RNF125 promote the ubiquitination and degradation of RIG-I (40, 53). Subsequently, we successfully screened RIG-I (**Figure 3**). Coincidentally, Kuniyoshi et al. (46) demonstrated that RIG-I can be activated by Mex3C. Therefore, we speculated that fish Mex3A may participate in RIG-I-mediated antiviral immune response just like mammalian Mex3C. RIG-I is usually located in the cytoplasm before virus infection (54). It was shown that RIG-I accumulates in stress granule (SG) to perform its antivirus function following viral infection (55). Furthermore, our results revealed that Mex3A and RIG-I are located in different regions within the cell under stimulation with poly(I:C). In particular, fish RIG-I was located in the late endosome (RAB7) after poly(I:C) stimulation, indicating that RIG-I can be transferred from the early endosome to the late endosome post-stimulation with poly(I:C) (**Figure S2**). Moreover, grass carp RIG-I was partially located in the mitochondria, and we suggest that this location of RIG-I is related to mitochondria protein MAVS. However, we found that CiMex3A was rarely located in the endosome (**Figure 4A**). The subcellular localization pattern is extremely different from Mex3B in mammals, because Mex3B was located in the early endosomes rather than in the endoplasmic reticulum (56).

Interestingly, both grass carp Mex3A and RIG-I share a partially similar location pattern in the endoplasmic reticulum. Further investigation is needed to determine whether Mex3A exerts its function together with RIG-I in the endoplasmic reticulum. We speculate that subcellular distributions of Mex3A and RIG-I are changed dynamically and are associated with their functions in the cytoplasm.

Previous studies have demonstrated that RIG-I is the target of many E3 ubiquitin ligases (40, 46). Our results suggested that Mex3A promotes the ubiquitination and degradation of RIG-I (**Figures 5C–F**), which is different from Mex3C-mediated K63-linked polyubiquitination in mammals (46).

It was reported that the tumorigenesis of human glioblastoma may be affected by Mex3A-induced ubiquitylation and degradation of RIG-I (57). Interestingly, grass carp Mex3A displayed a similar function in the ubiquitylation and degradation of RIG-I.

Mex3A truncation mutants (1–282 and 1–454) were mainly located in the cytoplasm, which is consistent with wild Mex3A (**Figure 6B**). Mex3A (1–282) or Mex3A (1–454) still possessed the same KH domain, which is a key domain that affects the distribution of Mex3 (26). In the TRIM family, the RING domain is reported to act as an essential E3 ubiquitin ligase domain responsible for substrate recognition (58). We found that Mex3A truncation mutant deleted in the RING domain fails to induce the ubiquitylation and degradation of RIG-I, suggesting that the RING domain is an indispensable part of fish Mex3A (**Figures 6D–F**).

## Data Availability Statement

The original contributions presented in the study are included in the article/**Supplementary Materials**. Further inquiries can be directed to the corresponding author.

## Ethics Statement

The animal study was reviewed and approved by Nanchang University.

## Author Contributions

CH supervised the research. ZJ conceived the study, and designed and performed the experiments. ZS, JH, and DL analyzed experimental data. XX, ML, ZF, SZ, and HM provided reagents, technical assistance, and contributed to the completion of the study. ZJ wrote the manuscript. All authors reviewed the results and approved the final version of the manuscript.

## Funding

This work was supported by research grants from the National Natural Science Foundation of China (32160871, 31960735, and 32160872), the Jiangxi Agriculture Research System (JXARS-06), the China Postdoctoral Science Foundation (2019M662279), and the Jiangxi Postdoctoral Science Foundation (2019KY43 and 2020RC22).

## Conflict of Interest

The authors declare that the research was conducted in the absence of any commercial or financial relationships that could be construed as a potential conflict of interest.

## Publisher’s Note

All claims expressed in this article are solely those of the authors and do not necessarily represent those of their affiliated organizations, or those of the publisher, the editors and the reviewers. Any product that may be evaluated in this article, or claim that may be made by its manufacturer, is not guaranteed or endorsed by the publisher.

## Supplementary Material

Supplementary Figure 1 Phylogenetic analysis of Mex3A. Phylogenetic tree was generated by Mega 6.0 program. The result was based on the multiple sequence alignment using the Clustal X program. The bars were 1000 assessments of bootstrap to determine the confidence level. CiMex3A was marked with black triangle.

Supplementary Figure 2 The location analysis of RIG-I. To further explore the location of RIG-I in cells. CIK cells were separately co-transfected with indicated plasmids. Then, the photos were taken under 63× oil immersion using Zeiss confocal microscope. The results are representatives of three independent experiments. The bars show 10 μm.
