# Supplementary figures and images for: Grass Carp Mex3A Promotes Ubiquitination and Degradation of RIG-I to Inhibit Innate Immune Response
Source: Front Immunol. 2022 Jul 5;13:909315. doi: 10.3389/fimmu.2022.909315 (PMC9295999; doi:10.3389/fimmu.2022.909315)

Figure S1 Phylogenetic analysis of Mex3A


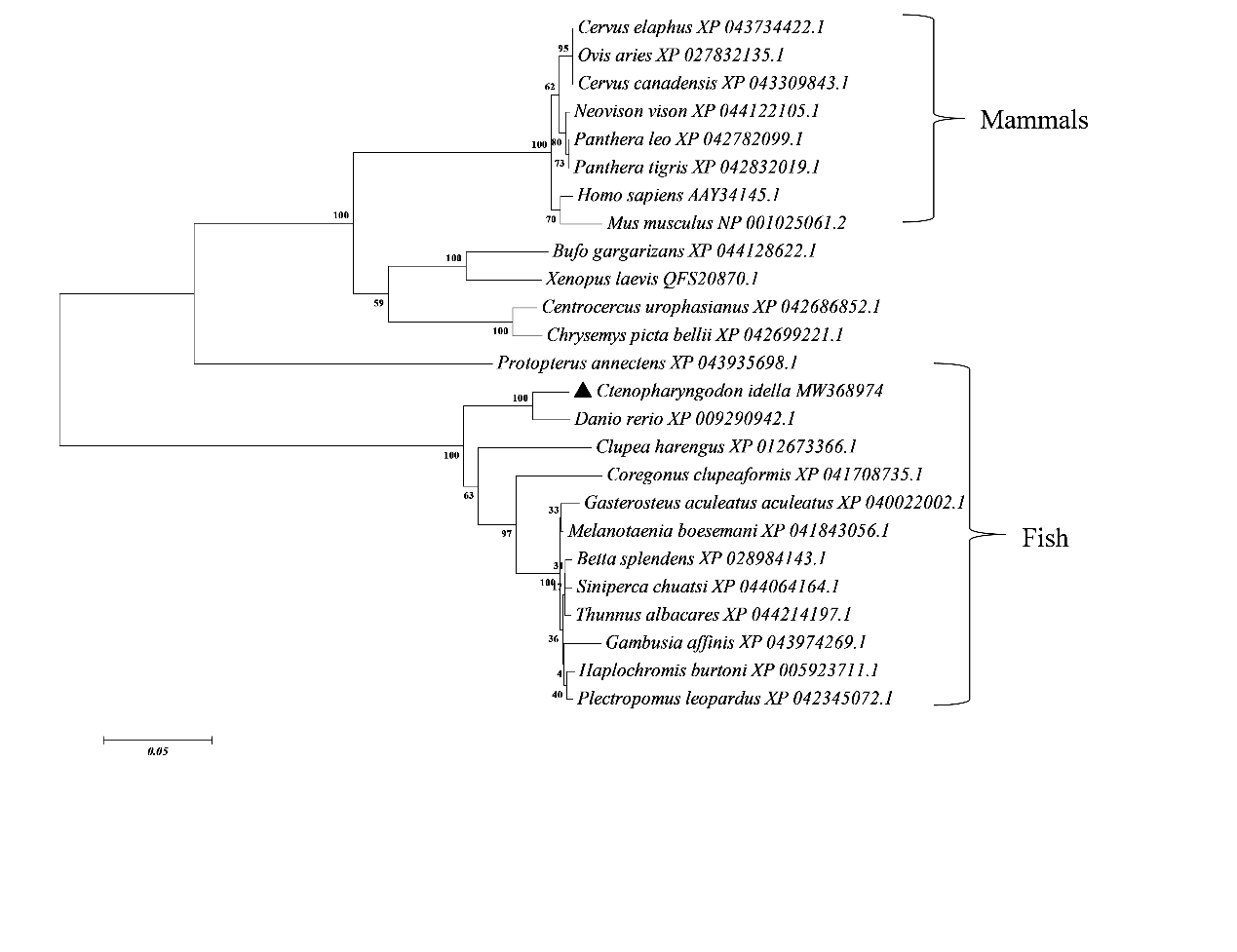


Figure S2 The location analysis of RIG-I


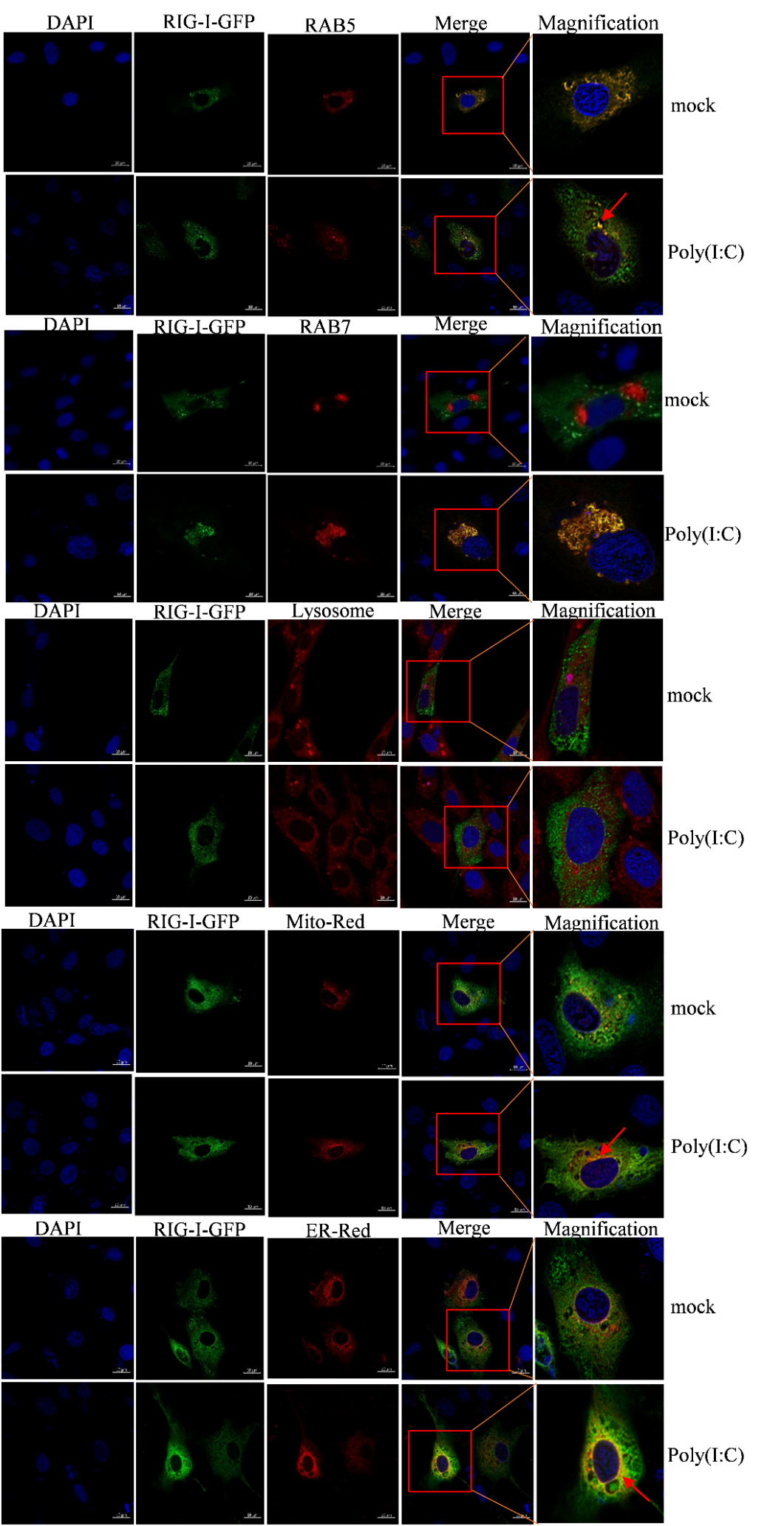

Supplement: Supplementary Figure 1 — Phylogenetic analysis of Mex3A. Phylogenetic tree was generated by Mega 6.0 program. The result was based on the multiple sequence alignment using the Clustal X program. The bars were 1000 assessments of bootstrap to determine the confidence level. CiMex3A was marked with black triangle. [file DataSheet_1.docx]
